# Antibiotic and heavy metal resistance of *Aeromonas hydrophila* and *Edwardsiella tarda* isolated from red hybrid tilapia (*Oreochromis* spp.) coinfected with motile aeromonas septicemia and edwardsiellosis

**DOI:** 10.14202/vetworld.2017.803-807

**Published:** 2017-07-21

**Authors:** S. W. Lee, W. Wendy

**Affiliations:** 1 Faculty of Agro Based Industry, Universiti Malaysia Kelantan Jeli Campus, 17600, Jeli, Kelantan, Malaysia; 2 Center for Fundamental and Liberal Education, Universiti Malaysia Terengganu, 21030 Kuala Nerus, Terengganu, Malaysia

**Keywords:** antibiotic, edwardsiellosis, heavy metal, motile aeromonas septicemia, multiple antibiotic resistance index, red hybrid tilapia

## Abstract

**Aim:**

The aim of this study is to identify antibiogram and heavy metal resistance pattern of *Aeromonas hydrophila* and *Edwardsiella tarda* isolated from red hybrid tilapia (*Oreochromis* spp.) coinfected with motile aeromonas septicemia and edwardsiellosis in four commercial fish farms.

**Materials and Methods:**

*A. hydrophila* and *E. tarda* were isolated using glutamate starch phenol red and xylose lysine deoxycholate (Merck, Germany) as a selective medium, respectively. All the suspected bacterial colonies were identified using conventional biochemical tests and commercial identification kit (BBL Crystal, USA). Susceptibility testing of present bacterial isolates to 16 types of antibiotics (nalidixic acid, oxolinic acid, compound sulfonamides, doxycycline, tetracycline, novobiocin, chloramphenicol, kanamycin, sulfamethoxazole, flumequine, erythromycin, ampicillin, spiramycin, oxytetracycline, amoxicillin, and fosfomycin) and four types of heavy metals (mercury, chromium, copper, and zinc) were carried out using disk diffusion and two-fold agar dilution method, respectively.

**Results:**

Three hundred isolates of *A. hydrophila* and *E. tarda* were successfully identified by biochemical tests. Antibiotic susceptibility testing results showed that 42.2% of the bacterial isolates were sensitive to compound sulfonamides, sulfamethoxazole, flumequine, oxytetracycline, doxycycline, and oxolinic acid. On the other hand, 41.6% of these isolates were resistant to novobiocin, ampicillin, spiramycin, and chloramphenicol, which resulted for multiple antibiotic resistance index values 0.416. Among tested heavy metals, bacterial isolates exhibited resistant pattern of Zn^2+^ > Cr^6+^ > Cu^2+^ > Hg^2+^.

**Conclusion:**

Results from this study indicated that *A. hydrophila* and *E. tarda* isolated from coinfected farmed red hybrid tilapia were multi-resistant to antibiotics and heavy metals. These resistant profiles could be useful information to fish farmers to avoid unnecessary use of antimicrobial products in the health management of farmed red hybrid tilapia.

## Introduction

Tilapia fish is the third most important aquaculture species in the world market where Asia becomes the main producer. Red hybrid tilapia is one of the popular freshwater species farmed intensively in Malaysia. This fish species has tasty flesh that drives demand from the market while its easy adaptation to new environment with fast growth attributes made it a preference species to fish farmers. Nevertheless, tilapia fish is susceptible to bacterial infections.

With the rapid expansion of aquaculture, Gram-negative bacteria *Aeromonas hydrophila* and *Edwardsiella tarda* have been recognized as the important pathogens responsible to many disease outbreaks in worldwide finfish farming. Disease caused by *A. hydrophila* and *E. tarda* is known as motile aeromonas septicemia (MAS) and edwardsiellosis, respectively. *A. hydrophila* and *E. tarda* were successfully isolated from moribund cage cultured silver catfish (*Pangasius sutchi*), striped catfish (*Pangasianodon hypophthalmus*), and red hybrid tilapia (*Tilapia* spp.) [1,2]. According to Zhang *et al*. [3], *A. hydrophila* caused significant economic loss in channel catfish, *Ictalurus punctatus* farming in the United States. There were also reports on *A. hydrophila* infection in other aquatic animals involving giant freshwater prawn, *Macrobrachium rosenbergii* [4] and American bullfrog, and *Rana catesbeiana* [5]. Edwardsiellosis frequently resulted in high mortality to the farmed fish when the fish were under stress due to poor husbandry and bad weather conditions [6].

It is well established in aquaculture industry that administration of antibiotics and heavy metals as therapeutic agents helps to promote growth and disease control in cultured fish. However, widespread use of antibiotics can lead to the development of antibiotic resistance in bacteria community while the presence of cumulative toxic metals is associated with the coselection mechanisms [7,8]. Therefore, this study focused in identifying the antibiotic and heavy metal susceptibility patterns of *A. hydrophila* and *E. tarda* in coinfected hybrid tilapia fish. Findings from this study will provide insight information to fish farmers when dealing with health management of MAS and edwardsiellosis coinfected fish.

## Materials and Methods

### Ethical approval

This study was conducted following approved guidelines of the Institutional Animal Ethics Committee. The members of the committee are comprised of researchers from Faculty of Agro Based Industry, Universiti Malaysia Kelantan Jeli Campus.

### Bacterial isolation and identification

**Figure-1:**
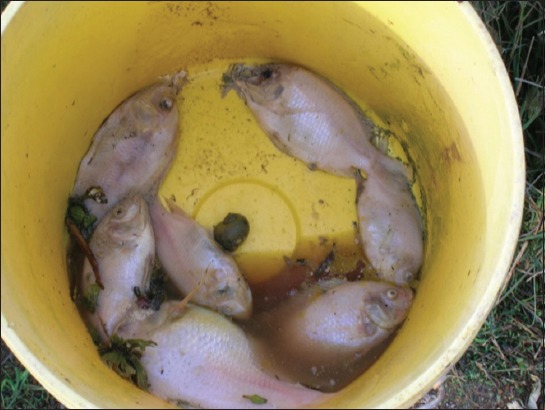
Sampled fish exhibited pigmentation loss, exophthalmia, eye opacity, and swelling at abdomen part.

Sampling of diseased fish (n=300) was carried out at four commercial fish farms located in Bachok, Pasir Puteh, Tanah Merah, and Jeli areas, Kelantan. The sampled fish weight from 200 to 350 g each with an exhibition of pigmentation loss, exophthalmia, eye opacity, and swelling at abdomen part (Figure-1). Bacterial isolation was carried out using glutamate starch phenol red (GSP) and xylose lysine deoxycholate (XLD) (Merck, Germany) as a selective medium. The fluid of internal and external of diseased fish was swabbed on the selective media. The inoculated media underwent incubation for 24-48 h at room temperature (25-28°C). All the suspected bacteria colony were identified using conventional biochemical tests [4] and followed with commercial identification kit (BBL, USA) [5].

### Antibiotic susceptibility test

Antibiotic susceptible test was performed using disk diffusion method [1]. The bacterial cells were prepared by propagated the bacteria using Luria Broth (Oxoid, England), followed by centrifugation at 14,500 rpm for 10 min using MiniSpin (Eppendorf, Germany). The collected bacterial cells were adjusted into 10^9^ colony forming unit (CFU) using physiological saline and monitored using Biophotometer (Eppendorf, Germany). The suspensions were swabbed on Mueller-Hinton agar (Oxoid, England) using sterile cotton bud. The inoculated media was then left for 10 min before antibiotic discs were placed onto the agar surface. 16 types of antibiotics tested were nalidixic acid (30 µg/disk), oxolinic acid (2 µg/disk), compound sulfonamides (300 µg/disk), doxycycline (30 µg/disk), tetracycline (30 µg/disk), novobiocin (30 µg/disk), chloramphenicol (30 µg/disk), kanamycin (30 µg/disk), sulfamethoxazole (25 µg/disk), flumequine (30 µg/disk), erythromycin (15 µg/disk), ampicillin (10 µg/disk), spiramycin (100 µg/disk), oxytetracycline (30 µg/disk), amoxicillin (25 µg/disk), and fosfomycin (50 µg/disk) (Oxoid, England). After 24 h of incubation period, diameter of the inhibition zone was measured in millimeter (mm) using a ruler and results were interpreted with reference to the standard provided by Clinical Laboratory Standards Institute [9].

### Determination of multiple antibiotic resistance (MAR) index

MAR index in this study was determined using the following formula:

Multiple antibiotic resistance (MAR) index = X/(Y × Z)

Where,

X = Total number of antibiotic resistance cases;

Y = Total number of antibiotics applied;

Z = Total number of bacteria isolates.

MAR index equal to or <0.2 indicated that the farmed fish were seldom or never exposed to the tested antibiotics; on the other hand, the farmed fish may have a high risk of exposure to the tested antibiotics if the MAR index is more than 0.2 [5].

### Heavy metal tolerance assay

These study bacteria isolates were subjected to heavy metals (mercury Hg^2+^, chromium Cr^6+^, zinc Zn^2+^, and copper Cu^2+^) tolerance assay through a series of two-fold agar dilutions method. The bacterial suspensions were prepared as described at a concentration of 10^9^ CFU. The bacterial suspensions were then spread onto the trypticase soy agar medium that incorporated with mercury II chloride, potassium dichromate, zinc sulfate, and copper II sulfate (Merck, Germany) in five different concentrations each. The concentrations for Cr^6+^ and Zn^2+^ were ranging from 25 to 400 μg/mL while the two-fold concentrations of Hg^2+^ and Cu^2+^ were ranging from 2.5 to 40 μg/mL and 150 to 2400 μg/mL, respectively. The inoculated media was incubated for 24 h at room temperature. The bacteria were considered resistant to the tested heavy metals if they grow at concentrations of 10 μg/mL Hg^2+^, 100 μg/mL for Zn^2+^ and Cr^6+^, and 600 μg/mLCu^2+^. The operational definition of tolerance as used in this study was based on positive bacterial growth when the concentration of heavy metals was above the stated concentration for resistance [10-12].

## Results

In this study, *A. hydrophila* (n=150) and *E. tarda* (n=150) were successfully isolated and identified by biochemical tests and Gram’s staining in which they were the dominant microbes that can be found in the external and internal organs of the diseased fish samples. The bacterial colonies with yellow in color on GSP medium were suspected as *A. hydrophila* while bacterial colonies with clear and black spot in center (size 1-2 mm) on XLD medium were suspected as *E. tarda*. *A. hydrophila* was found Gram-negative and non-swarming. They performed positive in oxidase and glucose-fermentative tests and were also found to utilize L-arabinose, D-lactose, D-mannose, D-mannitol, salicin, D-sorbitol, and sucrose. On the other hand, *E. tarda* that isolated in this study presented as Gram-negative, short rod, and positive in catalase test. These isolates can utilize glucose and fructose but failed to utilize lactose, trehalose, inositol, and sucrose. This result was supported by the symptoms and appearances of the diseased fish. In addition, the MAS and edwardsiellosis coinfected fish showed clinical signs of infection with the presence of nodules and abscesses in the spleen and kidney with cumulative mortality reached almost 10% from four sampling locations.

Overall, antibiotic susceptibility testing results showed that 42.2% of the bacterial isolates were sensitive to compound sulfonamides, sulfamethoxazole, flumequine, oxytetracycline, doxycycline, and oxolinic acid. On the other hand, 41.6% of these isolates were resistant to novobiocin, ampicillin, spiramycin, and chloramphenicol, which resulted for MAR index value 0.416. Intermediate sensitive cases were only 16.2%. *A. hydrophila* and *E. tarda* isolates were highly sensitive to compound sulfonamides (83.3%), followed by sulfamethoxazole (76.7%), flumequine (73.3%), oxytetracycline (72.0%), doxycycline (70.0%), and oxolinic acid (70.0%). On the other hand, bacteria isolates were highly resistant to novobiocin (78.0%), ampicillin (77.0%), spiramycin (74.7%), and chloramphenicol (74.0%) (Table-1). Among the four tested heavy metals, bacterial isolates exhibited resistant pattern of Zn^2+^ > Cr^6+^ > Cu^2+^ > Hg^2+^. The percentage of resistant to heavy metals ranged from 22.3% (Hg^2+^) to 67.3% (Zn^2+^) (Table-2).

**Table-1:** Susceptibility of *A. hydrophila* and *E. tarda* isolates from coinfected red hybrid tilapia to 16 types of antibiotics.

Antibiotic (µg/disk)	Resistant, R (%)	Intermediate sensitive, I (%)	Sensitive, S (%)
Nalidixic acid (30)	21.7	26.7	51.7
Oxolinic acid (2)	7.7	22.3	70.0
Compound sulfonamides (300)	9.7	7.0	83.3
Doxycycline (30)	4.0	26.0	70.0
Tetracycline (30)	58.0	25.3	16.7
Novobiocin (30)	78.0	11.3	10.7
Chloramphenicol (30)	74.0	7.7	18.3
Kanamycin (30)	68.3	15.0	16.7
Sulfamethoxazole (25)	15.7	7.7	76.7
Flumequine (30)	12.3	14.3	73.3
Erythromycin (15)	37.7	22.3	40.0
Ampicillin (10)	77.0	18.7	4.3
Spiramycin (100)	74.7	7.7	17.7
Oxytetracycline (30)	6.0	22.0	72.0
Amoxicillin (25)	69.7	11.3	19.0
Fosfomycin (50)	51.7	14.0	34.3

*A. hydrophila=Aeromonas hydrophila*, *E. tarda=Edwardsiella tarda*

**Table-2:** Heavy metal resistant profiles of *A. hydrophila* and *E*. *tarda* isolates from coinfected red hybrid tilapia.

Heavy metal	Resistant, R (%)
Hg^2+^	22.3
Cr^6+^	44.7
Zn^2+^	67.3
Cu^2+^	28.0

*A. hydrophila=Aeromonas hydrophila*, *E.tarda=Edwardsiella tarda*

## Discussion

MAS and edwardsiellosis are two bacterial diseases which mainly infected freshwater fish including red hybrid tilapia. Sudhesh *et al*. [13] reported that *A. hydrophila* was responsible to MAS infection in salmonid and non-salmonid fish, sturgeon, tilapia, catfish, striped bass, and eel, whereas *E. tarda* was claimed as the causative agent of edwardsiellosis in salmon, carps, tilapia, catfish, striped bass, flounder, and yellowtail. In this study, *A. hydrophila* and *E. tarda* which causing MAS and edwardsiellosis, respectively, were found to infect the sampled red hybrid tilapia in four commercial farms.

Compound sulfonamides performed as the most sensitive antibiotic in inhibiting the present bacterial isolates. In earlier, this antibiotic was found to be promising in controlling *Vibrio* spp. in Atlantic halibut, *Hippoglossus hippoglossus* through bath treatment [14]. However, antibiotic bath administration method is costly and not economically wise to be applied in red hybrid tilapia farming. Therefore, further study need to be carried out to apply this antibiotic orally. Besides, sulfamethoxazole, flumequine, oxytetracycline, doxycycline, and oxolinic acid were also found to be effective among the tested antibiotics in controlling the pathogens of this study. Oxolinic acid was also found to be effective in controlling edwardsiellosis first reported in cage cultured sharpsnout sea bream, *Diplodus puntazzo* from the Mediterranean [15]. Moreover, flumequine and oxolinic acid were quinolone antibiotics that were able to biodegrade at certain condition. Giraud *et al*. [16] claimed that application of oxolinic acid at the high concentration in an aquatic integrated farming does not affect the resistant level to the antibiotic among bacteria that isolated from sediments of the system. This finding was supported by the study of Lai and Lin [17] that quinolone antibiotic was found to biodegrade faster when exposed to light. Hence, oxolinic acid and flumequine would be a better choice of antibiotics to combat against MAS and edwardsiellosis in red hybrid tilapia in regard to the environment betterment. Doxycycline and sulfamethoxazole were also found to control up to 70% of the present bacterial isolates. These antibiotics may also be selected in managing red hybrid tilapia health. This in agreeable to studies by Phu *et al.*, [18] where doxycycline and sulfamethoxazole were applied in Vietnam aquaculture in controlling disease of striped catfish, *Pangasianodon hypophthalmus* with a suggestion of withdrawal period of 15 days for sulfamethaxazole [19].

Trace elements of zinc, copper, and chromium are essential for normal homeostasis and integrity of the animal host immune system, yet these heavy metal tend to persist in the environment and subsequently impact on the heavy metal tolerance of the bacteria and coselection of antibiotic resistance [7,8]. Moreover, microbial toxicity by zinc and copper can also be influenced by the solubility of the heavy metal compounds under physiological conditions [20]. In this study, *A. hydrophila* and *E. tarda* isolates were highly resistant to Zn^2+^ (67.3%) and most sensitive to Hg^2+^ (77.7%), where surrounding agriculture activities were likely to be the source of heavy metal contamination to the aquaculture system via soil and water. Bacteria isolated from various animal and environmental sources were found to survive in toxic heavy metals by possessing specific genetic mechanisms of resistance to these heavy metals [7,8]. Bacterial susceptibility to mercury in this study was in agreement to Hassen *et al*. [21] where mercury being the most toxic heavy metal to all tested bacteria, compared to copper, zinc, and chromium. On the other hand, Lima de Silva *et al*. [22] reported that bacteria from sewage can survive in Cr, Ag, and Hg by adaptation to the presence of heavy metal via extracellular barrier, active transport of metal ions, extracellular sequestration, intracellular sequestration, and reduction of metal ions mechanisms.

## Conclusion

Findings from this study demonstrated that *A. hydrophila* and *E. tarda* isolated from coinfected farmed red hybrid tilapia were multi-resistant to antibiotics and heavy metals. Excessive input of antibiotics and heavy metals may favor for the development of coselection and cross-resistance in the bacteria community of the aquaculture environment. Hence, fish farmers must be prudent in practicing health management and may consider natural products and vaccine as the alternative measures.

## Authors’ Contributions

SWL and WW conducted this study and analyzed the data of this study. Both authors contributed in the draft, revision and approval of the final manuscript.
